# Improving spatial localization in MEG inverse imaging by leveraging intersubject anatomical differences

**DOI:** 10.3389/fnins.2014.00330

**Published:** 2014-10-20

**Authors:** Eric Larson, Ross K. Maddox, Adrian K. C. Lee

**Affiliations:** ^1^Institute for Learning and Brain Sciences, University of WashingtonSeattle, WA, USA; ^2^Department of Speech and Hearing Sciences, University of WashingtonSeattle, WA, USA

**Keywords:** inverse imaging, magnetoencephalography, electroencephalography, source localization

## Abstract

Modern neuroimaging techniques enable non-invasive observation of ongoing neural processing, with magnetoencephalography (MEG) in particular providing direct measurement of neural activity with millisecond time resolution. However, accurately mapping measured MEG sensor readings onto the underlying source neural structures remains an active area of research. This so-called “inverse problem” is ill posed, and poses a challenge for source estimation that is often cited as a drawback limiting MEG data interpretation. However, anatomically constrained MEG localization estimates may be more accurate than commonly believed. Here we hypothesize that, by combining anatomically constrained inverse estimates across subjects, the spatial uncertainty of MEG source localization can be mitigated. Specifically, we argue that differences in subject brain geometry yield differences in point-spread functions, resulting in improved spatial localization across subjects. To test this, we use standard methods to combine subject anatomical MRI scans with coregistration information to obtain an accurate forward (physical) solution, modeling the MEG sensor data resulting from brain activity originating from different cortical locations. Using a linear minimum-norm inverse to localize this brain activity, we demonstrate that a substantial increase in the spatial accuracy of MEG source localization can result from combining data from subjects with differing brain geometry. This improvement may be enabled by an increase in the amount of available spatial information in MEG data as measurements from different subjects are combined. This approach becomes more important in the face of practical issues of coregistration errors and potential noise sources, where we observe even larger improvements in localization when combining data across subjects. Finally, we use a simple auditory N100(m) localization task to show how this effect can influence localization using a recorded neural dataset.

## Introduction

Magnetoencephalography (MEG) is a physiological measurement technique that provides millisecond resolution of neural activity recorded from hundreds of sensors simultaneously. Mapping MEG signals to the brain sources that generated them, however, is a difficult challenge because the number of sensors is far smaller than the number potential neural sources that give rise to the signals they record, and because electromagnetic field measurements could equivalently arise due to different source configurations even if additional sensors were available—the electromagnetic inverse problem is thus ill posed (Helmholtz, 1853). Thus, to localize MEG activity, additional constraints are necessary, where different methods are appropriate depending on underlying assumptions. For example, assuming that neural activity can be accounted for by a sparse constellation of focal sources or a set of distributed brain sources leads, respectively, to using minimum-current estimates (Gramfort et al., 2012) or minimum-norm estimates (Hämäläinen and Ilmoniemi, 1994). These issues result in ambiguity about which inverse method is appropriate, and what the underlying spatial resolution of MEG estimates is. With the increasing use of MEG for neuroimaging, addressing spatial specificity issues is especially important.

Although improving inverse approaches remains an active area of development (Gramfort et al., 2013b), here we approach the issue of MEG localization using the minimum-norm estimate (MNE), as this linear method remains a popular choice for MEG analysis. Minimum-norm approaches generally estimate neural activity to be of low-magnitude and broadly distributed among many potential sources, typically constrained to coming from cortical sources (Dale and Sereno, 1993), yielding current distributions that are spread out for even a focal underlying activation. The resulting “point spread” functions thus reflect the ambiguity of spatial localization using this method. In addition, there is cross-talk between nearby source locations that results in mixing of the actual brain activity if the distance between the activated areas is small (Liu et al., 2002).

Given this ambiguity and the challenges associated with MEG localization, MEG inverse imaging accuracy has been extensively investigated previously. For example, the choice of minimum-norm noise normalization has been shown to affect the resulting point-spread functions (Hauk et al., 2011), and minimum-norm procedures have been compared to other MEG localization methods in performance detecting signals in noise (Darvas et al., 2004). These and related studies are important because they investigate how well brain signals can be extracted from noisy recordings and localized. However, beneath these issues remains an important question regarding how accurately inverse methods can localize activity even under ideal conditions (i.e., no sensor or brain noise, perfect coregistration, etc.). This ideal case sets a bound on the spatial accuracy of MEG inverse imaging in practical applications—and this bound has thus far been widely assumed to be limited by the amount of spatial information available at the level of an individual subject.

It has been shown that MEG signals can be equivalently represented using an expansion based on spherical harmonics in a method called signal space separation (SSS; Taulu et al., 2005). This transformation, in addition to facilitating the elimination of environmental noise artifacts, is important because it has been used to show that the number of usable spatial components from MEG measurements asymptotes at around 100 components (Nenonen et al., 2007) even when several times more sensors are available (e.g., 306 for Elekta Neuromag systems). In other words, the number of useful spatial components in MEG data is limited to approximately 100, likely due to adequate spatial sampling of neuro-magnetic fields (Ahonen et al., 1993). SSS thus de-noises MEG data by projecting data into a reduced subspace, resulting in a reduced data rank. EEG, which measures the electric potentials on the scalp accompanying the magnetic fields measured using MEG, has similar physical constraints plus additional spatial smearing due to propagation through the scalp, so it may also have limited spatial information. Fortunately, MEG and EEG provide complementary measures. As a result their combination is beneficial, providing the best estimates of actual activity using non-invasive electrophysiological methods (Sharon et al., 2007; Molins et al., 2008). For simplicity, here we focus primarily on MEG source localization issues, but the results should directly generalize for M-EEG source localization (and potentially other inverse imaging approaches).

Even in the absence of noise, the underdetermined nature of the inverse problem and limited number of spatial components will thus limit how well brain activation can be localized in individual subjects using MEG inverse methods. This is considered by some in the neuroimaging community to be one of the primary restrictions of inverse MEG methods. The question thus arises: what is the limit for MEG inverse source estimation accuracy? Although inverse methods induce point spread functions (Liu et al., 2002) at an individual level, we hypothesize here that, due to anatomical differences between subjects, the point-spread functions of different subjects will only partially overlap—and critically overlap mainly around the true activation. In other words, if all subjects share the same activation at a given anatomical brain location, then combining data across subjects with differing brain geometry will lead to improved group-level localization compared to that of individual subjects, even in the absence of noise.

To test this, we acquired anatomical MR information alongside MEG sensor coregistration information for multiple subjects, and used standard methods to calculate an accurate forward solution to simulate MEG measurements resulting from arbitrary cortical activations. Such a simulation approach granted us access to “ground truth” for accurate localization across subjects that cannot be obtained in real-world experiments. We then used this ground truth to test how accurately a point source can be resolved using MEG minimum norm estimation, finding that combining information across subjects improves MEG source localization, even in the absence of noise. We also find that this improvement helps overcome inaccuracies in localization due to coregistration misalignment, a known source of error (Hillebrand and Barnes, 2003), as well as other potential confounds (e.g., choice of noise covariance, poor signal-to-noise ratio). Finally, we analyze data from subjects performing a simple auditory N100(m) (the magnetic counterpart to the N100 measured using EEG) localization experiment in order to provide evidence that the improvement observed is present beyond a synthetic dataset. As suggested by the modeling, the complementary spatial information sharpens localization as neural data is combined across subjects.

## Materials and methods

### Task

All human subjects gave informed consent according to procedures approved by the University of Washington Institutional Review Board. Twenty subjects (12 female, age 20–34 years) had normal hearing thresholds (20 dB or less at octave frequencies from 250 to 8000 Hz). They performed a 5-min task that required them to attend to brief auditory and visual stimuli, and detect infrequent oddballs. Stimuli consisted of a 1000 Hz tone (60 trials), downward sine sweep (10 trials; oddball), visual circle (60 trials), or visual square (10 trials; oddball). Auditory stimuli were delivered diotically at 65 dB SPL using sound-isolating tubal insertion earphones (Nicolet Biomedical Instruments) with digital-to-analog conversion and amplification (Tucker-Davis Technologies), and visual stimuli subtended 2.5 degrees, presented using PsychToolbox back-projected onto a screen 1 m from subjects using a PT-D7700U-K (Panasonic) projector. All four trial types (auditory/visual ×standard/deviant) were randomly interleaved with an inter-stimulus interval uniformly distributed between 1.3 and 3.3 s. Subject behavioral responses (button presses to deviants) were obtained using a button box.

### Anatomical information

Structural MRI data were collected with a 3T Philips scanner. One standard structural multi-echo magnetization-prepared rapid gradient echo scan and two multi-echo multi-flip angle (5° and 30°) fast low-angle shot scans, each approximately 8 min, were acquired from (passive) subjects. In a separate session, a 3Space Fastrak (Polhemus) was used to register the spatial locations of cardinal landmarks (nasion, left/right periauriculars), four head position indicator (HPI) coils, and additional points on the scalp. These were used to coregister MEG sensors with the structural MRI. Subjects were then placed in the MEG scanner (306-channel system, Elekta Neuromag) and the location of the subject's head relative to the MEG sensors was continuously reported by the HPI coils while subjects performed the oddball detection task.

Using the anatomical MR scans, a cortical source space was constructed using dipoles with 7 mm spacing, yielding roughly 3500 dipoles per hemisphere normal to the cortical surface located along the gray/white matter boundary segmented from the MRI (Dale et al., 1999) using Freesurfer (http://surfer.nmr.mgh.harvard.edu/). Note that this is fewer than the 20,484 dipoles on the average brain template, ensuring that upsampling to the common template would not lose spatial information. Combining this structural information with subject colocation information, a three-compartment boundary element model (BEM) was used to provide an accurate calculation of the forward solution (Mosher et al., 1999) mapping dipole currents in the brain (source space) to observable M/EEG signals (sensor space).

### Source estimation

M/EEG signals were used to estimate dipole currents in the brain using the anatomically constrained minimum-norm linear estimation approach. A rich literature has been built over the last two decades focused on how this approach works; see e.g., Dale and Sereno (1993) and Hämäläinen and Ilmoniemi (1994) for details. Briefly, given a set of sensor readings **Y**, sensor covariance matrix **C**, forward solution **G**, and the regularization parameter λ, if the source covariance matrix **R** is identity (i.e., no *a priori* source weighting) as it is here, the minimum-norm reconstruction of neural sources **X**_MNE_ is given by:

$$X_{\text{MNE}}=RG^{T}{\left(GRG^{T}+\lambda C\right)}^{-1}Y=G^{T}{\left(GG^{T}+\lambda C\right)}^{-1}Y$$

The typical interpretation of **C** in this method is that it represents the spatial covariance of noise to be suppressed in source estimation resulting from sensor noise and/or baseline brain state. To process data from the neural experiment, **C** was estimated from 200 ms epochs prior to each stimulus onset to capture non-task-related brain noise. To process data from simulations, **C** was estimated from an empty room noise covariance matrix recorded after subjects exited the MEG once the task was complete. We used the empty-room noise covariance matrix in simulations to eliminate any task-related source localization biases that could be introduced. This prevented us from easily incorporating EEG in simulations, but results from the MEG simulations should generalize in a straightforward manner to M/EEG measurements.

To preprocess the empty room data, data were band-pass filtered between 1 and 40 Hz, and six spatial components (four for magnetometers, two for gradiometers) were projected out using signal space projection (SSP) to help suppress potential noise sources (Uusitalo and Ilmoniemi, 1997). To process recorded neural data, we band-passed between 1 and 10 Hz [the frequency range of interest likely to carry N100(m) information] and made use of SSS (Uusitalo and Ilmoniemi, 1997) to suppress any environmental artifacts. This gave rise to three choices of noise covariance: (1) from the empty room recording (ERM), (2) empty room with SSS, and (3) during the baseline of the task (also with SSS); as such we tested the effects of using these different noise covariances on localization accuracy. In cases where SSS was performed, 10 inner components and 2 outer components were chosen based on the quality of the recorded data.

Although there was no noise in most of our simulated brain activity (see *Activity simulation and localization accuracy quantification*, below), a regularization parameter of λ = 1/9 (corresponding to an estimated SNR of 3, as SNR = √(1/λ)) was used in all source reconstructions, unless otherwise noted. This standard parameter choice is the default for the MNE software package (Gramfort et al., 2013a, 2014) and was used to help capture the amount of spatial point spread observed in typical experiments. In one set of simulations, we also specifically tested the effect of this parameter on localization accuracy.

To compensate for resulting sensitivity differences across cortex and non-uniformities in point spread, the standardized low resolution tomography (Pascual-Marqui et al., 1994; Pascual-Marqui, 2002) form of the minimum-norm solution was used. sLORETA can be thought of as one particular form of MNE, as it is linearly related to MNE by a simple spatial normalization in source space based on the diagonal entries of the whitened resolution matrix (**X**_MNE_**G**) as:

$$X_{\text{sLORETA}}=\left(\text{diag}{\left(\text{diag}\left(X_{\text{MNE}}\left[C+\lambda^{-2}GRG^{T}\right]X_{\text{MNE}}^{T}\right)\right)}^{-0.5}\right)X_{\text{MNE}}$$

This makes sLORETA closely related to other minimum-norm-based linear inverse methods (Mosher et al., 2003). We use sLORETA for all source localization here (despite the fact that sLORETA may not achieve zero-bias localization at high noise levels), but we expect that other noise-normalizing solvers could likely also be used to achieve similar effects.

Source localization data were mapped from individual subjects to an average brain using a standard non-linear spherical morphing procedure designed to optimally align sulcal-gyral patterns across subjects; for details see Fischl et al. (1999) and Gramfort et al. (2014). Data from each subject's low-resolution source space were morphed into the high-resolution cortical surface of the “fsaverage” brain using the MNE software's built-in iterative smoothing procedure (Gramfort et al., 2014). Briefly, this procedure first spreads activity to neighboring vertices on the cortical surface using an isotropic diffusion process with one parameter (the number of smoothing steps), then uses Freesurfer's registration algorithm to map between high-resolution surfaces of the two subjects, and finally downsamples to the destination source space. For simulations we used 5 steps of this procedure, as this is sufficient to fill the high-resolution surface during morphing. For neural data activation maps, we used 25 smoothing steps to help compensate for inter-subject functional variability (which is not otherwise present by design in our synthetic dataset).

### Activation simulation and localization accuracy quantification

To quantify the accuracy of a spatial localization across cortex, we examined how well a given point source commonly activated across subjects could be localized. To simulate activity, for each source location on the “fsaverage” average brain (containing 20,484 dipoles), the closest vertex for each subject based on spherical morphing was activated. Outside of the activated vertex of interest, the rest of the brain source activations were set to zero. Setting all other brain activation to zero was done to remove confounds of source noise modeling and subsequent statistical approaches. These procedures depend on considerations such as noise structure and signal-to-noise ratio, which are explored below in a subset of additional simulations (see *Introducing sources of error and noise*, below). Noise was also omitted from most simulations to ensure any observed localization improvements would not be due to simple signal denoising. After simulating activity at a given spatial location, we projected the activity to MEG sensors using the forward solution to simulate neural activity that could be recorded from a subject with a controlled activation pattern. Then, an inverse solution was used to map the MEG activations back to the source (brain) space, as would be done in a typical minimum norm approach. Finally, individual subject activations were mapped to the average brain using spherical morphing.

With MEG activations for each subject projected onto the average brain, we then sought to quantify the quality of the source localization by measuring both the activity centroid estimation and the point spread when using one subject's or the average of several subjects' activations to localize activity. To approximate the outcome of any given statistical approach—which would need to be selected based on the number of subjects used and the noise structure, among other things, in a non-simulated scenario—we selected the *V* points with the largest sum of activation magnitudes across subjects. We used different values of *V* as 1, 2, 5, 10, 25, 50, or 100 vertices as a surrogate for how many points a given statistical test could ideally recover from background noise, which is analogous to choosing a particular threshold on the activity level to declare points “significant.” With these *V* points selected, we calculated the Euclidean distance from the spatial centroid of these points to the true, original activation location (thereby quantifying the accuracy; see Figures 2A–C), as well as the average distance from each of the *V* points to the original activation location (thereby quantifying the point spread; see Figures 2D–F). Note that, since we simulate activity at a single source vertex, activation at any other vertex would constitute a false positive according to signal detection theory; our point spread measure thus quantifies the spatial distribution across cortex of the false positives that arise from the inverse solution. For a given desired subject count *N* (spanning from one to the number of subjects recorded from), the centroid estimation error and point-spread were determined by using the average over 50 random sub-samplings of the subjects of size *N* (with 50 iterations chosen to reduce computation time while providing sufficiently smooth estimates as a function of space and number of subjects). In our plots of centroid error and point-spread (Figures 2A,C) we have masked the inner portion of the medial wall, since this may contain structures (corpus callosum, midbrain) with unreliable MEG sensitivity and localization.

In these simulations, the physical simulation is linear (sources add linearly to produce activation maps), as are the inverse operators and the smoothing operators, and our scoring functions operate on the maximally active *V* points. These factors make it so the overall simulated amplitude of each source location is arbitrary—the results would be the same given any linear scaling of all source vertex activations. Activation amplitudes used can and should thus be thought of as being on an arbitrary scale.

### Introducing sources of error, noise, and variability

There are many factors that affect M/EEG source localization, and the simulations described above examine combining spatial information across subjects in a somewhat idealized setting. For example, they have infinite signal-to-noise ratio (SNR) and the coregistration is perfect. There are also factors such as the regularization parameter λ and the noise covariance used **C** that, while are common choices for analysis, vary across studies and affect source localization accuracy. Given that these factors (among others) may affect localization accuracy, we explored each of them in different subsets of simulations. For simplicity, each subset of simulations analyzed just the top *V* = 25 points instead of parametrically varying the parameter, since the *V*-curve family in the standard simulation all followed the same trends (see Results). These potential issues are described one-by-one in the following paragraphs.

First, note that the standard simulations above use identical forward solutions in the simulation of brain activity and generation of the inverse solution. In real experiments, it is not possible to achieve this level of coregistration (and corresponding forward solution) accuracy. To test the effect of misalignment in coregistration, we repeated the above analysis, again testing localization accuracy when combining data across subjects. This time, we tested different spatial “shifts” between the coregistration transformation used to simulate the data, and the transformation used to generate the forward solution used in inverse estimation. In order to replicate the procedure from a previous investigation into this issue (Hillebrand and Barnes, 2003), we used shifts of 0 (no difference), 2, 4, and 8 mm between the two forward solutions. To simulate a given shift, a random number was drawn from a normal distribution with zero mean and standard deviation equal to the shift parameter (2, 4, or 8 mm). The magnitude of this number was then used as the distance from true coregistration (with the direction randomly selected from a uniform distribution). This process results in the distances being effectively drawn from a folded normal distribution, where the original shift parameters (2, 4, and 8 mm) yield expected values for distances of 1.60, 3.19, and 6.38 mm, respectively. This range of coregistration errors should capture the misalignment expected in real experiments, with errors using fiducial and scalp digitization estimated to range from 1.3 to 4.4 mm (Whalen et al., 2008). This procedure was repeated 10 times for each subject to simulate different coregistration errors. Because errors in coregistration effectively amount to gross errors in forward solution estimation, testing different degrees of coregistration error can be considered as a surrogate for testing different levels of inaccuracy in forward solution modeling (e.g., arising from an inaccurate BEM).

Second, some of our simulations used a noiseless brain (SNR = ∞; only activating the target brain vertex) to ensure that resulting localization gains would be due to increased spatial information as opposed to effects of signal de-noising. However, it is also informative to see the extent to which these improvements can persist in the face of noise, so we ran a set of simulations parametrically varying the SNR. To do this, we introduced Gaussian noise at the sensor level with spatial structure equal to that observed in the baseline of the oddball task. This captures the spatial structure of both the sensor (thermal) and brain (resting-state) “noise” that experimenters typically seek to suppress in experiments, allowing us to quantify how the SNR (−10, 0, or 10 dB) affects combining data across subjects. Here SNR is defined as 20 times the log_10_ ratio of the amplitude of the evoked signal and the evoked noise.

Third, we examined the issue of noise covariance used in inverse estimation. Since our standard simulated data used an empty-room (ERM) noise covariance, we tested how using a task-derived baseline noise covariance would affect localization. Since the data recorded from participants (which was used to estimate the task-based covariance) was processed using SSS, we also ran a condition where we processed the ERM noise covariance with SSS.

Finally, in our standard simulations we made use of the minimum-norm inverse regulation parameter λ = 1/9 (thought of as estimated an SNR of 3, where λ is typically conceptualized as being related to the estimated SNR, as SNR = λ^−1/2^) because it is a fairly standard choice in MEG studies. However, we also tested different regularization parameters corresponding to estimated SNR 0.3, 1, 3, 10, or 30 to examine the effects on localization.

## Results

### Localization accuracy increases when adding subjects

**Figure 1:**
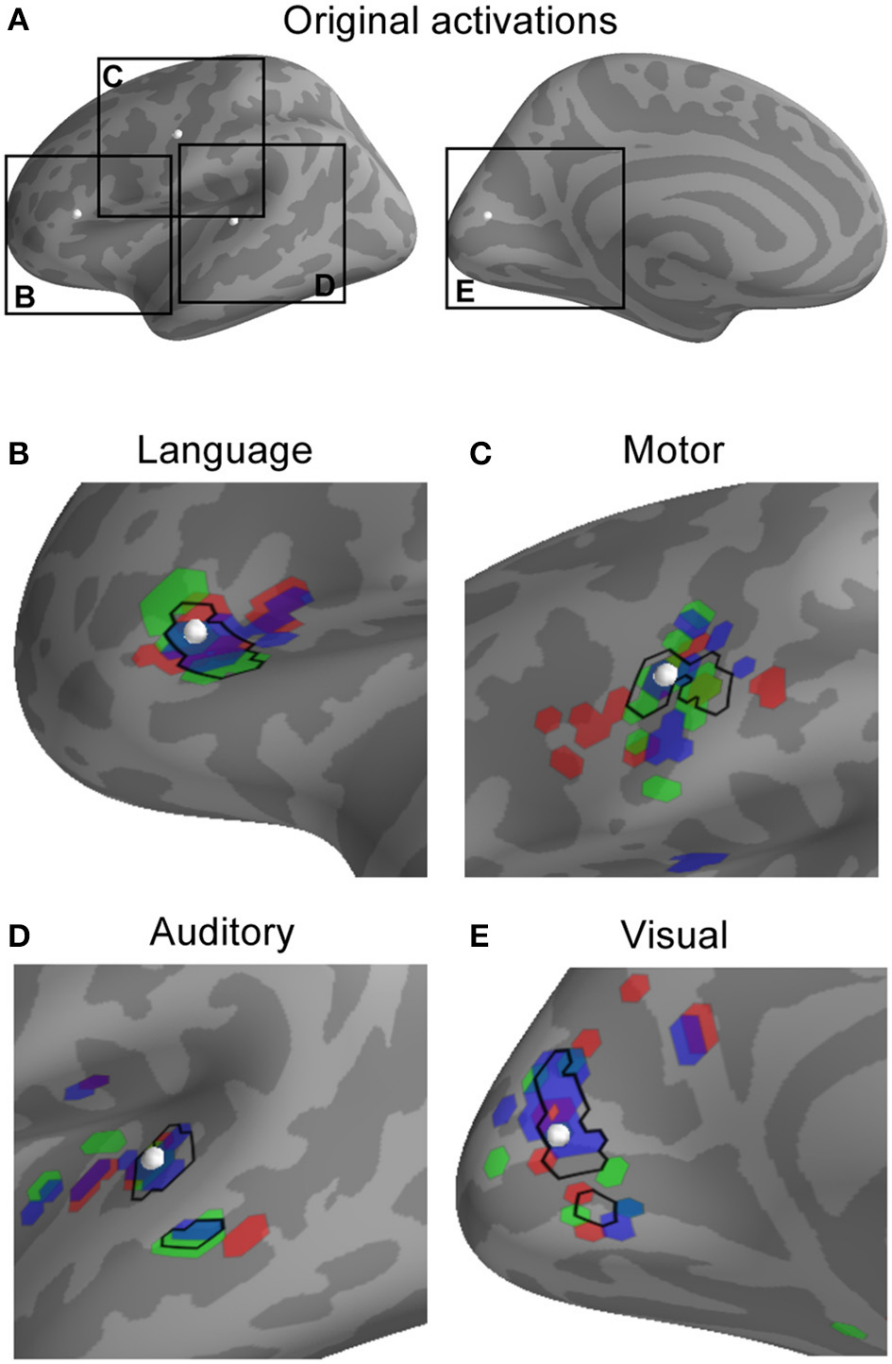
**Observed activity patterns resulting from simulated activation in representative locations in language, motor, auditory, and visual areas**. The location of each simulated active dipole is shown (white spheres in **A**) as well as individual activation patterns **(B–E)**. For each location, the source activation was projected into the MEG sensor space (as potentially observable data) and then the minimum-norm inverse solution with spherical morphing was used to map the activity to a common brain. In the plot for each region (language, motor, auditory, visual), the top 25 most active points for three different sample subjects are shown in red, green, and blue, alongside the top 25 most active points for the average across all subjects (black outline). Note that the point-spread functions—indicated by activity that has moved or even jumped sulci or gyri away from the location of underlying true activity—differ between subjects, and that the average across subjects is converging toward the point of original activation.

Anatomical MRI, MEG coregistration information, task-related and empty-room recordings were obtained from 20 subjects in order to examine how combining MEG inverse imaging estimates across subjects can affect localization of the neural activity. First, we simulated point-source activity from the cortical surface of each subject, with the location of activation conserved across subjects, and examined how precisely and accurately the inverse solution localized the average activity across subjects. Figure 1 shows an example of the activity simulation for four different point-source dipole activations. Note that while the individual subjects' point-spread patterns differ for each region, they do share some common spatial overlap which, crucially, includes the location of the true activation.

**Figure 2:**
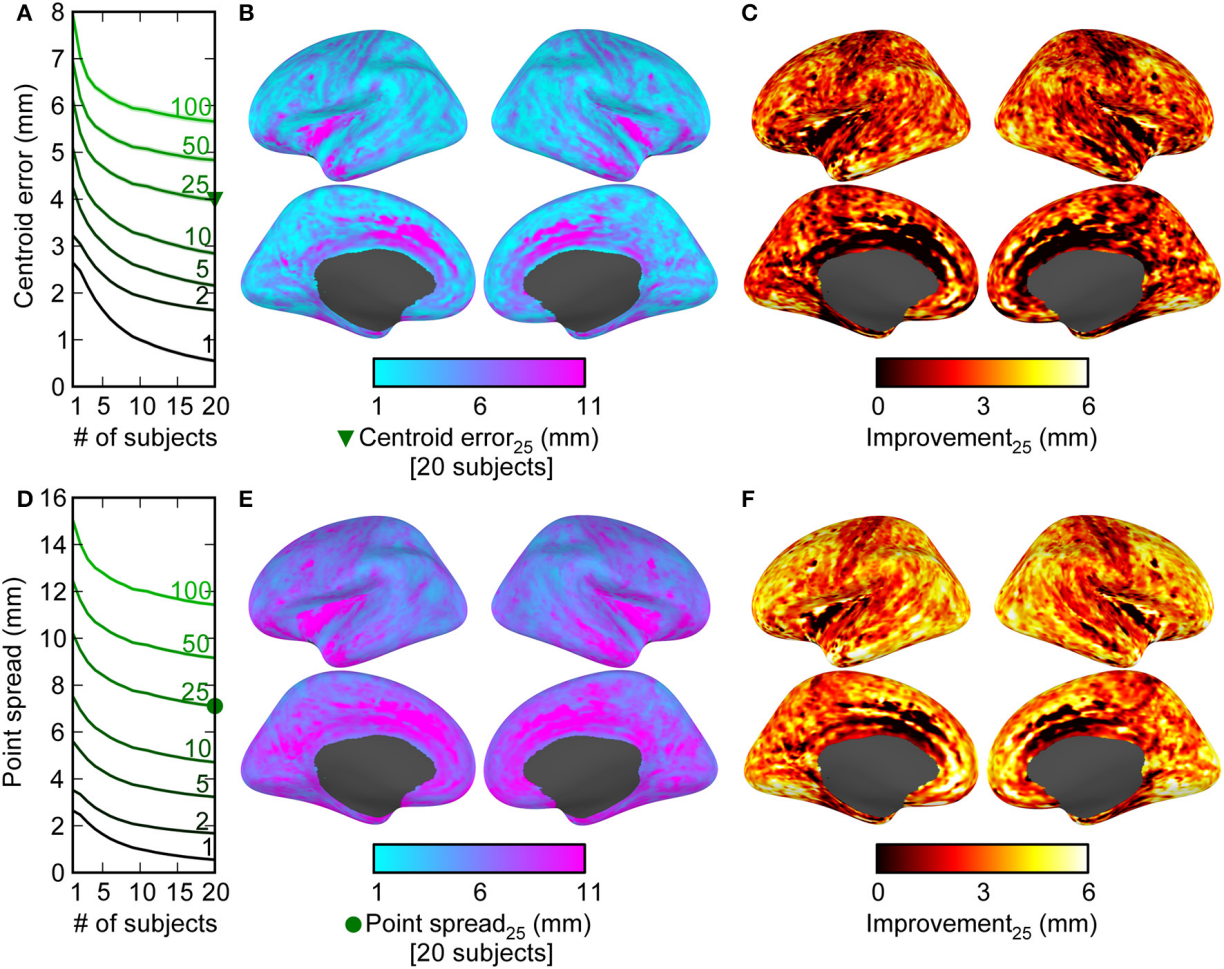
**Combining information across subjects results in decreases in both the error in the estimation of centroid location (accuracy), and the point-spread of the data (precision)**. The centroid error **(A–C)** was measured by the difference between the true activation location and the centroid calculated from the top *V* points (averaged across spatial locations for *V* = 1, 2, 5, 10, 25, 50, or 100 in *A*; *V* = 25 for **B,C**) across different numbers of subjects (1 through 20). The point spread **(D–E)** was similarly measured as the average distance between the location of true activation and each of the top *V* spatial locations. Mean ± 2 s.e.m. (across 20,424 cortical locations) is shown by the lines and shaded backgrounds. Note that s.e.m. values are mostly small enough to be masked by the mean lines. The improvement due to combining data across subjects as a function of cortical location is shown in **(B,C,E,F)**. There were large decreases across cortex when comparing 20 subjects to 1 in both the centroid estimation error **(C)** and the point-spread **(F)**, indicating that the differing subject anatomical structure has reduced the spatial uncertainty and reduced localization error. Note also that the best absolute error values (e.g., some near 1 mm) are only valid for ideal conditions.

We quantified the localization accuracy in two ways. The first was the centroid estimation error, which we calculated as the difference between the location of the original activation and the centroid calculated from the top *V* points from the average activation across *N* subjects. The second was the point-spread, which quantified the precision by taking the average distance from the true activation point and each of the top *V* points from the average activation across subjects. For any given number of *V* points spanning 1, 2, 5, 10, 25, 50, or 100 vertices, we observed substantial reductions in both centroid estimation error and point-spread comparing data from 20 subjects to data from one subject (Figure 2). The average centroid localization error and point-spread are shown averaged across space for different numbers of “significant” points *V* (Figures 2A,D) and different numbers of subjects used. The localization error and point-spread are displayed for *V* = 25 points across the cortex to show the spatial pattern of spatial localization quality for including all 20 subjects (Figures 2B,E) and the difference between all 20 subjects and one subject (Figures 2C,F). While the best absolute error values (e.g., some around 1 mm) are likely not reflective of values that can be obtained in a real experiment (due to several factors not simulated in this figure), the localization differences between using multiple subjects and using a single subject show that complementary information across subjects can lead to improved spatial localization. Examining the spatial pattern of both the gains from adding subjects and the raw values for using 20 subjects suggest that medial areas have the lowest localization accuracy and gain the least from averaging across subjects. It also appears as though the improvement is greater for radial sources (tops of gyri, bottoms of sulci) compared to tangential sources.

### Compensating for errors and noise

**Figure 3:**
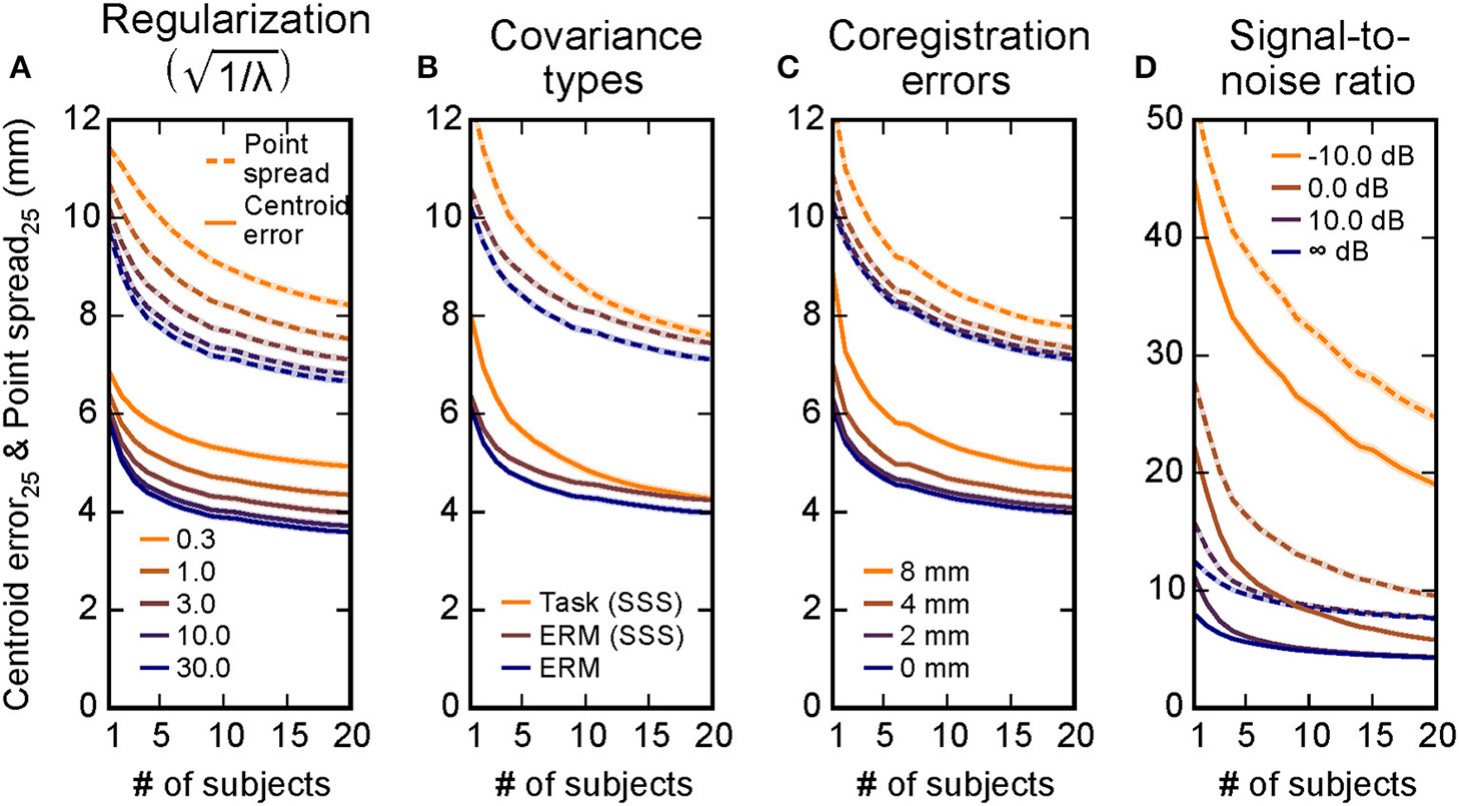
**Combining information across subjects compensates for inaccuracies in localization due to multiple experimental factors**. Simulations were run covering different regulation parameters λ **(A)**, noise covariances used in inverse estimation **(B)**, errors in coregistration alignment **(C)**, and a range of signal-to-noise ratios **(D)**. The centroid error (solid lines) and the point spread (dashed lines) were both reduced as information across subjects was combined in all of these scenarios, suggesting that the simulation results from Figure 2 generalize to many situations. Mean ± 2 s.e.m. (across 20,424 cortical locations) is shown by the lines and shaded backgrounds. Note that s.e.m. are mostly small enough to be masked by the mean lines; standard deviation values across cortex are provided in Table 1. Here for simplicity only the *V* = 25 point case is shown, the *V* = 25 lines in Figures 2A,D equivalent here to the $\sqrt{\left(1/\lambda \right)}=3$ case in A, the ERM case in B, and the 0 mm case in C. The infinite SNR case in D corresponds to the task-based covariance case from B, since the evoked covariance was used in the inverse solutions for that simulation.

We then sought to quantify the extent to which combining data across subjects can compensate for deleterious effects of errors, noise, and other factors that exist in real experiments. We examined the effect of combining data across subjects across five different manipulations to introduce different forms of “noise” by:

1. Varying the regularization parameter λ (related to the experimenter-estimated SNR as λ = SNR^−2^; Figure 3A).
2. Using three different noise covariance matrices (Figure 3B).
3. Inducing coregistration errors consistent with a previous study (Hillebrand and Barnes, 2003): spatial shifts of 0 (no error), 2, 4, and 8 mm between the “true” coregistration and the one used to estimate the anatomically constrained inverse solution (Figure 3C).
4. Adding noise to the simulations with realistic sensor-plus-“brain noise” spatial structure derived from the baseline period in the oddball task (Figure 3D).

In each of these five cases, we again found that combining data across subjects improves source localization (Figure 3). For example, for errors in coregistration, the average accuracy across spatial locations when combining data across 20 subjects compared to 1 subject goes from 6.1 mm to 4.0 mm in the shift = 0 mm case and 9.0 mm to 5.1 mm in the shift = 8 mm case, improvements of 34 and 44%, respectively. The only factor that induced a large shift from the general results from the original, unmodified simulations (Figures 2A,B, *V* = 25 case) were the localization errors introduced by signal-to-noise ratio. For these data, combining data across subjects greatly reduces the localization error and point spread. The changes from using one subject to using all 20 subjects for each condition are summarized in Table 1.

**Table 1:** **Performance improvement due to combining data across subjects under various simulated changes**.

		**CE_1_**	**CE_20_**	**PS_1_**	**PS_20_**	**ΔCE(%)**	**ΔPS(%)**
**Lambda [λ^−1/2^]**	**0.3**	6.9 ± 1.8	4.9 ± 3.0	11.4 ± 1.9	8.2 ± 2.7	**28.2**	**28.1**
	**1**	6.4 ± 1.7	4.4 ± 2.6	10.7 ± 1.8	7.5 ± 2.3	**32.3**	**29.7**
	**3**	6.1 ± 1.6	4.0 ± 2.3	10.2 ± 1.7	7.1 ± 2.0	**35.1**	**30.4**
	**10**	6.0 ± 1.6	3.7 ± 2.2	9.9 ± 1.8	6.8 ± 1.9	**37.6**	**31.2**
	**30**	5.9 ± 1.6	3.6 ± 2.2	9.7 ± 1.8	6.7 ± 2.0	**38.9**	**31.6**
**Covariance (type)**	**Task (SSS)**	8.0 ± 3.4	4.3 ± 3.3	12.5 ± 3.5	7.6 ± 3.5	**46.8**	**39.2**
	**ERM (SSS)**	6.4 ± 1.8	4.2 ± 2.5	10.6 ± 1.9	7.4 ± 2.1	**33.5**	**29.8**
	**ERM**	6.1 ± 1.6	4.0 ± 2.3	10.2 ± 1.7	7.1 ± 2.0	**35.1**	**30.4**
**Coreg. error (mm)**	**0**	6.1 ± 1.7	4.0 ± 2.3	10.2 ± 1.7	7.1 ± 2.0	**35.1**	**30.3**
	**2**	6.3 ± 1.7	4.1 ± 2.4	10.4 ± 1.7	7.2 ± 2.1	**35.5**	**30.6**
	**4**	7.0 ± 1.6	4.3 ± 2.4	10.9 ± 1.7	7.3 ± 2.1	**38.8**	**32.7**
	**8**	8.9 ± 1.6	4.9 ± 2.5	12.5 ± 1.7	7.8 ± 2.2	**45.6**	**37.8**
**SNR (dB)**	**-10**	44.9 ± 12.2	19.0 ± 18.9	52.0 ± 13.3	24.7 ± 21.6	**57.7**	**52.5**
	**0**	22.2 ± 11.2	5.8 ± 5.5	27.7 ± 12.5	9.5 ± 6.7	**74.0**	**65.7**
	**10**	11.2 ± 4.8	4.3 ± 3.3	15.8 ± 5.3	7.7 ± 3.6	**61.1**	**51.2**
	**8**	8.0 ± 3.4	4.3 ± 3.3	12.5 ± 3.5	7.6 ± 3.5	**46.5**	**39.0**

Centroid error is CE (mm), point spread is PE (mm), and the Δ values are given by, e.g., ΔCE = 100*(CE_1_ − CE_20_)/CE>_1_ (percent). Each measure is shown ± 1 standard deviation across cortical locations.

### Real use example: N100(m) localization

**Figure 4:**
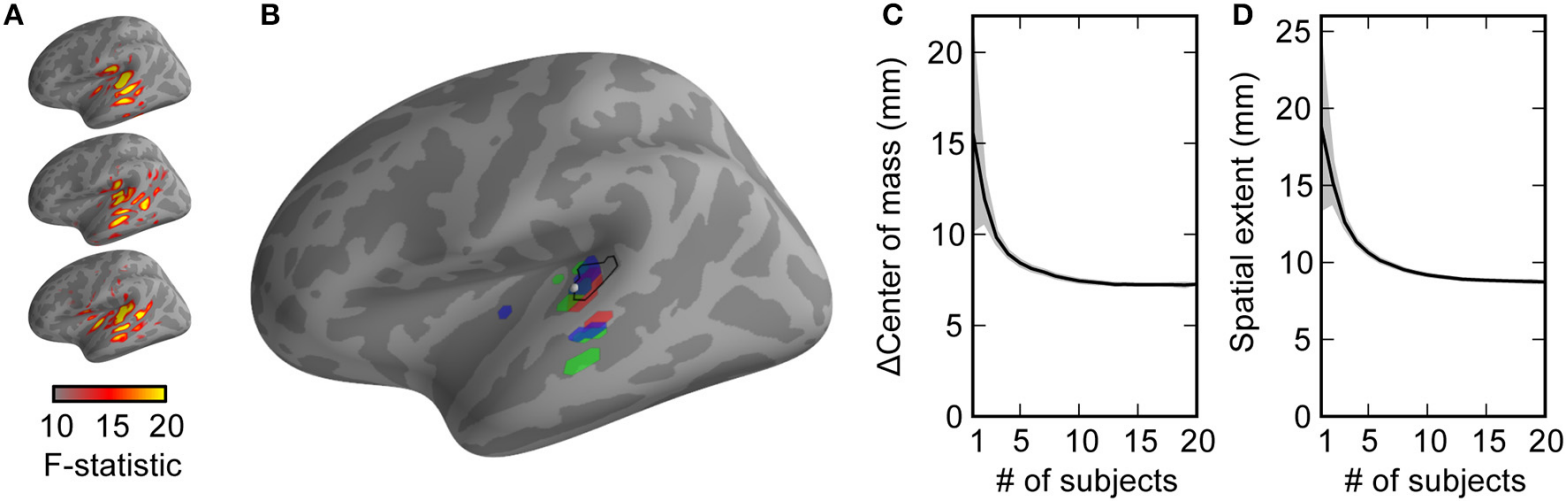
**In a simple real experiment, auditory N100(m) localization improves as data are combined across subjects, consistent with modeling predictions**. **(A)** The activation maps (F-statistic values from sLORETA) for multiple subjects show activation in primary auditory areas, as well as point spread activation due to M/EEG inverse imaging. **(B)** The highest-activation (most statistically significant) points from each subject (colors) are more scattered than the highest-activation points from the average across subjects (black). The difference between the center of mass and an estimate of established N100(m) localization **(C)** and the spatial extent of the activation **(D)** is shown as a function of subjects. Mean ± 2 s.e.m. (across up to 2000 different random combinations of subjects) is plotted by the lines and shaded backgrounds. Note that these values were calculated the same way as the “centroid error” and “point spread” from simulations, but the lack of a ground truth activation location (or extent) made these labels inappropriate to assign to the ordinate axes.

We sought to test these principles using a simple auditory oddball-detection task while recording M/EEG data from subjects. The neural activity occurring 100 ms post-stimulus in multiple subjects tend to include standard areas, such as Heschl's gyrus and planum temporale; see Figure 4A for activations from three example subjects. While the estimated activation extends beyond these anatomical regions—due at least in part to the point-spread functions induced by M/EEG inverse imaging—note that each subject's activation map has a slightly different spatial pattern. This is readily apparent when selecting the top *V* = 25 most active vertices for these sample subjects and comparing them activations to those of the average across the combination of all 20 subjects (Figure 4B). Although N100(m) generator localization varies from study to study, Figure 4B shows a location estimate for the N100(m) generator approximated based on previous work (Gutschalk et al., 2007; Saenz and Langers, 2014). Quantifying how much the centroid estimation differs from this location, as well as the spatial extent of the activation pattern (Figure 4C) shows a sharpening and localization improvement when data are combined across all 20 subjects, consistent with the modeling results.

## Discussion

### Improvement in localization across subjects

Many modern neuroimaging experiments are predicated on the notion that there is a set of underlying patterns of brain activity that are conserved across different subjects who participate in a given experimental paradigm. When researchers attempt to measure such common activation patterns from a set of subjects, inter-subject variability typically complicates the process of finding any true underlying similarities in activation. Here we investigated the extent to which inter-subject variability in anatomical information could paradoxically improve source localization accuracy of MEG. We hypothesized that differences in individual subject anatomy would create differences in point-spread functions that would increase the accuracy and precision of source localization, since the point-spread functions would overlap consistently in the location of true activation and inconsistently elsewhere. For a given focal source activation common across a set of subjects, our results strongly suggest that this is indeed the case, with estimates of activation location and extent being decreased when data from more subjects are used. This suggests that MEG minimum-norm inverse solutions, which are designed to provide a distributed estimate of source localization, can be combined across subjects to improve localization estimates. In other words, the random variations in spatial brain geometry across subjects can actually improve spatial localization of activations that are conserved across a population.

It is important to note that spatial improvements in real experiments can be affected by a number of factors. Here we simulated specifically how multiple such factors affect spatial localization. In terms of overall trends in localization for a single subject (not considering changes as data are combined across subjects), first we found that the inverse regularization parameter λ increased the point spread with decreasing signal-to-noise prior estimate (Figure 3A). This was to be expected due to the mathematics behind the regularization (which essentially set a prior on the smoothness). Second, we found only minor differences between empty-room covariances that had or had not been processed using SSS, with larger differences for the localization using a covariance from the task-related baseline period (Figure 3B). This, too, is wholly expected for the simulation, since the task-related covariance will necessarily suppress some brain activity (i.e., that from regions active during the baseline) that was simulated and subsequently localized. Third, we found a degradation of localization performance due to shifts in coregistration from the ideal location (Figure 3C), similar to what was found in a previous study (Hillebrand and Barnes, 2003). Finally, we also showed by adding noise to the simulations that the SNR of the signal to be detected can have quite a large impact on localization (Figure 3D). In this case, adding subjects not only added spatial information, but also played an important role in de-noising the data.

Critically, we show here that combining data across subjects not only has a strong ameliorative effect on many of these factors and errors, but that the benefit often increases with the severity of a given error. For example, in the covariance case, using a covariance that actually includes activation from areas of interest should hurt localization. In our simulations, this occurs because the task-related covariance necessarily contains some of the simulated spatial locations (e.g., increasing from about 6 to 8 mm error)—yet combining data across subjects reduces the localization error introduced so that the accuracy of the data combined across 20 subjects is nearly equivalent in both cases. There is a similar effect for coregistration errors, where combining data across subjects helps rescue the group-level localization. Notably, coregistration inaccuracies are one particular failure mode of forward modeling, and thus our results indicate that similar effects may occur in situations with other forward modeling issues. For example, certain errors in forward modeling can result in around 4 mm localization error (Ramon et al., 2006), similar to the amount we observed as a result of an 8 mm shift in coregistration. Although we tested multiple factors, our list was certainly not exhaustive, and there are likely other factors that affect localization performance. Nevertheless, while these issues will predictably decrease overall localization performance, they may serve to enhance (as opposed to diminish) the gains provided by combining data across subjects. Given these considerations, our results demonstrate that complementary spatial anatomical information across subjects can, sometimes drastically, improve spatial localization in MEG inverse estimates.

We next showed that localization can improve using a neural dataset collected from 20 subjects performing a basic oddball-detection task. Our N100(m) localization results (Figure 4) were within the level of variability observed in previous work (Gutschalk et al., 2007), and show a clear trend of tightening of the source estimation as data are combined across subjects. Of course, these data must be interpreted carefully. In these data, the “ground truth” for location is unknown, so that the difference in center of mass (Figure 4C) asymptotes to the difference between our study and the reference ones (Gutschalk et al., 2007; Saenz and Langers, 2014). Additionally, assuming that more than a single point-source within each subject's brain was active during the task, the extent of activation (Figure 4D) should converge to some non-zero value. However, the concordance between these results that are based on neural data and those yielded by our simulations suggest that the simulated observations translate to real scenarios.

Although we only tested localization improvement using real data for auditory cortex N100(m) localization, Figure 2 suggests that the localization improvements should be observed over most areas in cortex. Examining the spatial pattern of localization quality, we observed a lack of accurate localization and limited gain from adding additional subjects in the case of medial regions (Figure 2). We speculate that this is due to the hemispheric ambiguity in detecting medial activity using MEG. Specifically, the proximity of mirrored parallel structures makes estimating the hemisphere of activation challenging in those regions, and this will not be helped much by adding additional subjects. It is also possible that the deeper medial sources are not as well localized by the MEG sensors. In addition, the differences in improvements between radial vs. tangential sources suggest that the actual cortical folding also plays a role in the improvement, and it is possible that the folding in medial regions is not as variable across subjects, thereby offering a smaller advantage when averaging across subjects.

### Practical considerations

All of our simulations make use of single dipole-like activations (spatial δ functions) to test localization accuracy. This was because it provided access to the ground truth—namely a single location with no point spread—and because single points of activation should pose a worst-case for minimum-norm localization (which assumed distributed activation). An important issue that our study does not directly address is the fact that neural activity during a given task tends to involve multiple co-activated regions with (potentially) broad activations. Properly localizing broad activations and detecting two or more simultaneously active regions (and differentiating between them) is a difficult problem. However, this challenge may lie more in the statistical domain than that of inverse imaging—given the linearity of the physical activations being measured and the linearity of the inverse, detecting the centroid of one source, or detecting one source vs. multiple activations becomes dependent on the ability to pull relevant signals out of the noise and identify (or perhaps “cluster”) them appropriately. At the inverse imaging end, there is an issue of how many independent spatial components exist when combining data across subjects—and our data suggest there is more spatial information than typically thought. It nonetheless remains unclear which of these factors will provide an upper bound on the number of resolvable sources, and a full investigation of these issues would be valuable. It would also be useful to fully examine how different sizes, shapes, and forms of broader activation patches would affect the ability to localize the active sources.

Here we have also made use of a simple metric (the top *V* active points) as a surrogate for a statistical test, since we did not need to compensate for sources of noise beyond those introduced by using an empty-room noise covariance. We tested multiple values of number of points to use *V* as a proxy for different statistical thresholds, and saw consistent trends in improvements. The present results will likely influence broad issues regarding which statistical tests to use and how many subjects are necessary in a given study, as well as more specific ones such as how best to define spatial clusters in non-parametric tests under a given subject count. In real-world use, noise structure, and signal-to-noise variability both within a given subject (across both time and space), and across subjects can have important implications on which statistical technique is appropriate, and how focal the resulting estimates are (Genovese et al., 2002; Nichols and Holmes, 2002; Pantazis et al., 2005). Moreover, activity location variability across subjects within a given task was not explored here, and it is beyond the scope of the current investigation to determine the extent to which non-overlapping point-spread functions facilitate localization in this scenario. However, activity location variability is a confound that all neuroimaging studies must address (including those with higher spatial precision such as fMRI), and thus is not specific to MEG inverse imaging; this is likely a cause of the increased popularity of measures such as multi-voxel pattern analysis (Norman et al., 2006).

Our work also presumes that spherical morphing provides an ideal mapping from one subject's brain anatomy to another. While it is possible that a different form of intersubject mapping would yield better (or worse) results in real-world applications, this can also be thought of as a form of improper cross-subject variability compensation. The appropriate deployment of statistical approaches, compensating for cross-subject underlying activity location variability, and investigation of optimal cross-subject mappings could all serve as useful venues for subsequent investigations, but will not likely eliminate potential impact of increasing spatial information by combining information across subjects. Although we have chosen one specific workflow in our study (e.g., sLORETA inverse, Freesurfer transformations, simple activation measure), the results ought not to hinge on this—the increases in spatial information will persist across many different methods, and the specific measures carried out here help show the potential resulting effect in simulations and recorded data.

### Why localization improves

We speculate that the observed improvements in localization are facilitated by increased spatial information of MEG data in the common space. Because the initial simulations (e.g., Figures 1, 2) had no noise or induced inaccuracies (e.g., incorrect coregistration), it is unclear where else the improved localization could have come from. The fact that these improvements persisted in the face of noise then suggest that they will persist in real scenarios.

An interesting question thus arises regarding where additional spatial information comes from. It likely comes predominantly, if not entirely, from the differences in anatomical information between subjects. Underlying anatomical structures can give rise to different point-spread functions, as differences in geometry imply differences in dipole orientation, which (along with the sensor arrangement) determine the point-spread. Thus what is typically a challenging source of variability—intersubject differences in experiments—can, perhaps counterintuitively, improve spatial resolution for anatomically-constrained minimum-norm inverse imaging. Projecting data from sensor space onto source space using anatomical information could thus be thought of as providing a high-dimensional space where complementary spatial information can be meaningfully combined. How the topological properties of different subject anatomical structures can give rise to different point-spread functions that may lead to improved localization is left as an open question. It also remains to be seen the extent to which these results would generalize to other inverse methods, such as adaptive beamformers or minimum-current estimates. However, this benefit will not exist in studies where across-subject data are averaged in sensor space, or where a non-individualized head model is employed.

### Conflict of interest statement

The authors declare that the research was conducted in the absence of any commercial or financial relationships that could be construed as a potential conflict of interest.

## References

[B1] Ahonen A. I. Hamalainen M. S. Ilmoniemi R. J. Kajola M. J. Knuutila J. E. T. Simola J. T.. (1993). Sampling theory for neuromagnetic detector arrays. IEEE Trans. Biomed. Eng. 40, 859–869. 8288276 10.1109/10.245606

[B2] Dale A. M. Fischl B. Sereno M. I. (1999). Cortical surface-based analysis. I. Segmentation and surface reconstruction. Neuroimage 9, 179–194. 9931268 10.1006/nimg.1998.0395

[B3] Dale A. M. Sereno M. I. (1993). Improved localization of cortical activity by combining EEG and MEG with MRI cortical surface reconstruction: a linear approach. J. Cogn. Neurosci. 5, 162–176. 23972151 10.1162/jocn.1993.5.2.162

[B4] Darvas F. Pantazis D. Kucukaltun-Yildirim E. Leahy R. M. (2004). Mapping human brain function with MEG and EEG: methods and validation. Neuroimage 23(Suppl. 1), S289–S299. 10.1016/j.neuroimage.2004.07.014 15501098

[B5] Fischl B. Sereno M. I. Dale A. A. (1999). Cortical surface-based analysis. II: inflation, flattening, and a surface-based coordinate system. Neuroimage 9, 195–207. 10.1006/nimg.1998.0396 9931269

[B6] Genovese C. R. Lazar N. A. Nichols T. (2002). Thresholding of statistical maps in functional neuroimaging using the false discovery rate. Neuroimage 15, 870–878. 10.1006/nimg.2001.1037 11906227

[B7] Gramfort A. Kowalski M. Hämäläinen M. (2012). Mixed-norm estimates for the M/EEG inverse problem using accelerated gradient methods. Phys. Med. Biol. 57, 1937–1961. 10.1088/0031-9155/57/7/1937 22421459 PMC3566429

[B8] Gramfort A. Luessi M. Larson E. Engemann D. A. Strohmeier D. Brodbeck C.. (2013a). MEG and EEG data analysis with MNE-Python. Front. Neurosci. 7:267. 10.3389/fnins.2013.00267 24431986 PMC3872725

[B9] Gramfort A. Luessi M. Larson E. Engemann D. A. Strohmeier D. Brodbeck C.. (2014). MNE software for processing MEG and EEG data. Neuroimage 86, 446–460. 10.1016/j.neuroimage.2013.10.027 24161808 PMC3930851

[B10] Gramfort A. Strohmeier D. Haueisen J. Hämäläinen M. S. Kowalski M. (2013b). Time-frequency mixed-norm estimates: sparse M/EEG imaging with non-stationary source activations. Neuroimage 70, 410–422. 10.1016/j.neuroimage.2012.12.051 23291276 PMC3615257

[B11] Gutschalk A. Oxenham A. J. Micheyl C. Wilson E. C. Melcher J. R. (2007). Human cortical activity during streaming without spectral cues suggests a general neural substrate for auditory stream segregation. J. Neurosci. 27, 13074–13081. 10.1523/JNEUROSCI.2299-07.2007 18045901 PMC6673394

[B12] Hämäläinen M. S. Ilmoniemi R. J. (1994). Interpreting magnetic fields of the brain: minimum norm estimates. Med. Biol. Eng. Comput. 32, 35–42. 8182960 10.1007/BF02512476

[B13] Hauk O. Wakeman D. G. Henson R. (2011). Comparison of noise-normalized minimum norm estimates for MEG analysis using multiple resolution metrics. Neuroimage 54, 1966–1974. 10.1016/j.neuroimage.2010.09.053 20884360 PMC3018574

[B14] Helmholtz H. (1853). Ueber einige Gesetze der Vertheilung elektrischer Ströme in körperlichen Leitern mit Anwendung auf die thierisch-elektrischen Versuche. Ann. Phys. 165, 211–233

[B15] Hillebrand A. Barnes G. R. (2003). The use of anatomical constraints with MEG beamformers. Neuroimage 20, 2302–2313. 10.1016/j.neuroimage.2003.07.031 14683731

[B16] Liu A. K. Dale A. M. Belliveau J. W. (2002). Monte Carlo simulation studies of EEG and MEG localization accuracy. Hum. Brain Mapp. 16, 47–62. 10.1002/hbm.10024.abs 11870926 PMC6871820

[B17] Molins A. Stufflebeam S. M. Brown E. N. Hämäläinen M. S. (2008). Quantification of the benefit from integrating MEG and EEG data in minimum l2-norm estimation. Neuroimage 42, 1069–1077. 10.1016/j.neuroimage.2008.05.064 18602485

[B18] Mosher J. C. Baillet S. Leahy R. M. (2003). Equivalence of linear approaches in bioelectromagnetic inverse solutions, in 2003 IEEE Workshop on Statistical Signal Processing, 294–297. 10.1109/10.245606

[B19] Mosher J. C. Leahy R. M. Lewis P. S. (1999). EEG and MEG: forward solutions for inverse methods. IEEE Trans. Biomed. Eng. 46, 245–259. 10097460 10.1109/10.748978

[B20] Nenonen J. Taulu S. Kajola M. Ahonen A. (2007). Total information extracted from MEG measurements. Int. Congr. Ser. 1300, 245–248. 10.1016/j.ics.2007.01.058

[B21] Nichols T. E. Holmes A. P. (2002). Nonparametric permutation tests for functional neuroimaging: a primer with examples. Hum. Brain Mapp. 15, 1–25. 10.1002/hbm.1058 11747097 PMC6871862

[B22] Norman K. A. Polyn S. M. Detre G. J. Haxby J. V. (2006). Beyond mind-reading: multi-voxel pattern analysis of fMRI data. Trends Cogn. Sci. 10, 424–430. 10.1016/j.tics.2006.07.005 16899397

[B23] Pantazis D. Nichols T. E. Baillet S. Leahy R. M. (2005). A comparison of random field theory and permutation methods for the statistical analysis of MEG data. Neuroimage 25, 383–394. 10.1016/j.neuroimage.2004.09.040 15784416

[B24] Pascual-Marqui R. D. (2002). Standardized low-resolution brain electromagnetic tomography (sLORETA): technical details. Methods Find. Exp. Clin. Pharmacol. 24(Suppl. D), 5–12. 12575463

[B25] Pascual-Marqui R. D. Michel C. M. Lehmann D. (1994). Low resolution electromagnetic tomography: a new method for localizing electrical activity in the brain. Int. J. Psychophysiol. 18, 49–65. 7876038 10.1016/0167-8760(84)90014-x

[B26] Ramon C. Haueisen J. Schimpf P. H. (2006). Influence of head models on neuromagnetic fields and inverse source localizations. Biomed. Eng. Online 5, 55. 10.1186/1475-925X-5-55 17059601 PMC1629018

[B27] Saenz M. Langers D. R. M. (2014). Tonotopic mapping of human auditory cortex. Hear. Res. 307, 42–52. 10.1016/j.heares.2013.07.016 23916753

[B28] Sharon D. Hämäläinen M. S. Tootell R. B. H. Halgren E. Belliveau J. W. (2007). The advantage of combining MEG and EEG: comparison to fMRI in focally stimulated visual cortex. Neuroimage 36, 1225–1235. 10.1016/j.neuroimage.2007.03.066 17532230 PMC2706118

[B29] Taulu S. Simola J. Kajola M. (2005). Applications of the signal space separation method. IEEE Trans. Signal Process. 53, 3359. 10.1109/TSP.2005.853302

[B30] Uusitalo M. A. Ilmoniemi R. J. (1997). Signal-space projection method for separating MEG or EEG into components. Med. Biol. Eng. Comput. 35, 135–140. 9136207 10.1007/BF02534144

[B31] Whalen C. Maclin E. L. Fabiani M. Gratton G. (2008). Validation of a method for coregistering scalp recording locations with 3D structural MR images. Hum. Brain Mapp. 29, 1288–1301. 10.1109/10.748978 17894391 PMC6871211

